# Pituitary macroadenoma: less common neuroimaging features in a common lesion

**DOI:** 10.1590/0100-3984.2025.0065

**Published:** 2025-12-15

**Authors:** Fernanda Veloso Pereira, Natália Yaktine Yoshida, Davi Ferreira Soares, Heraldo Mendes Garmes, Denise Engelbrecht Zantut-Wittmann, Fábio Rogério, Mateus Dal Fabbro, Juliana Ávila Duarte, Fabiano Reis

**Affiliations:** 1 Faculdade de Ciências Médicas da Universidade Estadual de Campinas (FCM-Unicamp), Campinas, SP, Brazil; 2 Pontifícia Universidade Católica de Campinas (PUC-Campinas), Campinas, SP, Brazil; 3 Instituto Tecnológico da Aeronáutica (ITA), São José dos Campos, SP, Brazil; 4 Hospital de Clínicas de Porto Alegre (HCPA), Porto Alegre, RS, Brazil

**Keywords:** Adenoma, Pituitary neoplasms, Neuroendocrine tumors, Magnetic resonance imaging., Adenoma, Neoplasias hipofisárias, Tumores neuroendócrinos, Ressonância magnética.

## Abstract

Pituitary neuroendocrine tumors, previously known as pituitary adenomas, are the most common pituitary gland tumors; when larger than 10 mm in diameter, they are called pituitary macroadenomas. Although most pituitary macroadenomas exhibit characteristic imaging features, some present with uncommon neuroimaging manifestations. Less common imaging manifestations include hemorrhage, areas of necrosis, cystic components, calcifications, bone invasion, and extrasellar location. Knowing how to recognize the atypical neuroimaging patterns of pituitary macroadenomas is also crucial for identifying the potential aggressive behavior of the lesion. In addition to predicting aggressive behavior, recognition of atypical neuroimaging characteristics can help the neurosurgeon determine the most effective surgical approach. The aim of this review was to highlight the less common imaging patterns of pituitary macroadenomas.

## Abstract (pt)

Os adenomas hipofisários são os tumores mais comuns da glândula pituitária e, quando maiores que 10 mm, são chamados de macroadenomas. Os macroadenomas tipicamente exibem características de imagem características, mas podem se apresentar menos frequentemente com manifestações de neuroimagem incomuns. Manifestações de imagem menos comuns incluem hemorragias e áreas de necrose, componentes císticos, calcificações, invasão óssea e localização extrasselar. Saber reconhecer os padrões atípicos de neuroimagem dos macroadenomas hipofisários também é crucial para identificar o potencial de comportamento agressivo da lesão. Além do comportamento agressivo, as características atípicas de neuroimagem podem ajudar o neurocirurgião a determinar a abordagem cirúrgica mais eficaz. Esta revisão tem como objetivo destacar os padrões de imagem menos comuns dos macroadenomas hipofisários.

## INTRODUCTION

Pituitary neuroendocrine tumors (PitNETs), previously known as pituitary adenomas, account for approximately 10% of all brain tumors and approximately one-third to one-half of tumors found in the sellar and parasellar regions^(1)^.

According to the 2021 World Health Organization (WHO) classification, PitNETs are now classified on the basis of the adenohypophyseal cell lineages, rather than solely on the basis of the hormone produced^(2)^. When larger than 10 mm in diameter, they are referred to as pituitary macroadenomas. Most pituitary tumors (10-20%) are asymptomatic and are often found incidentally during the investigation of other medical issues^(1)^. In one review of the literature, Tahara et al.^(3)^ found that the frequency of pituitary incidentalomas ranged from 1.5% to 31.1%.

A slowly enlarging pituitary macroadenoma increases the size of the bony sella and protrudes into the suprasellar cistern. These tumors, when typical, often exhibit a “figure-of-eight” or “snowman” shape due to the inflexible dura of the diaphragm sellae creating a constricted middle section in the mass^(4)^.

Some PitNETs exhibit uncommon characteristics, suggesting a higher risk of aggressive behavior. Not all tumors exhibiting these different morphological characteristics display aggressive behavior; therefore, the term “atypical adenoma” was removed from the 4th edition of the WHO classification in 2017^(5)^.

A less common imaging pattern seen in pituitary macroadenomas is extrasellar extension, mainly into the suprasellar cistern, cavernous sinus, and sphenoidal sinus; other less common imaging findings include cystic components, hemorrhage, necrotic areas, calcifications, and bone invasion^(6-9)^. Depending on their location and hormonal activity, pituitary macroadenomas can provoke a variety of symptoms, including visual disturbances and hormonal imbalances^(10)^. Nonsecreting pituitary macroadenomas typically extend or grow into the cavernous sinus less often than do secreting ones^(6)^.

The aim of this study was to review and illustrate the atypical imaging features of pituitary macroadenomas.

## DISCUSSION

Pituitary adenomas are monoclonal tumors from the adenohypophysis and, according to the Central Brain Tumor Registry of the United States, constitute the second most common primary tumor of the central nervous system and the most common tumor of the adenohypophysis. For nomenclature, a cutoff point of 10 mm is used; they are referred to as pituitary microadenomas when smaller than 10 mm and as pituitary macroadenomas if greater than or equal to 10 mm^(11)^.

Tumor shapes were classified as ovoid, snowman-like, or (superiorly or inferiorly) lobulated. A snowman shape was defined as a figure of eight shape and as a superiorly or inferiorly lobulated shape with two or more lobes in the suprasellar or sellar compartments, respectively^(5)^. A pituitary macroadenoma can grow and affect nearby structures, frequently expanding upward into the suprasellar cistern, which causes compression of the optic chiasm or optic nerves. It can also spread downward into the sphenoid sinus and extend backward into the dorsum sellae, as well as invading the cavernous sinuses on the sides in some cases^(6)^.

Computed tomography (CT) is useful for detecting pituitary tumors that lead to enlargement of the sella turcica, having been proven effective in up to 94% of cases. On CT scans, most pituitary macroadenomas have attenuation similar to that of the pituitary gland on unenhanced CT and show substantial enhancement after contrast administration. It is essential to note that CT can reveal bone alterations caused by the expanding lesion, such as sellar enlargement, which may lead to sellar erosion or remodeling^(6)^.

Magnetic resonance imaging (MRI) is the preferred imaging technique for diagnosing PitNETs, and high spatial resolution images are essential for a proper assessment^(12)^. The protocol for MRI includes thin slices and a small field-of-view before and after contrast use (dynamic and static sequences). According to the Brazilian College of Radiology and Imaging Diagnosis^(13)^, the mandatory sequences are axial T2-weighted imaging (T2WI) or axial fluid attenuated inversion recovery of the skull; coronal T1WI and T2WI; coronal dynamic T1WI during and after intravenous injection of gadolinium contrast; and sagittal T1WI before and after intravenous administration of gadolinium. The high spatial resolution must have a slice thickness of ≤ 4 mm with an interslice gap of ≤ 4 mm. Additional sequences, such as diffusion-weighted imaging (DWI) and fluid attenuated inversion recovery, can be included. Utilizing T2*-WI gradient-echo MRI is currently the most effective neuroimaging method for detecting brain hemorrhage. Techniques such as T2*WI and susceptibility-weighted imaging (SWI) are susceptible to the paramagnetic effects of deoxyhemoglobin and methemoglobin, with bleeding products and hemosiderin deposits appearing as areas with markedly low signal intensity^(14)^.

On MRI, pituitary macroadenomas typically appear slightly hypointense or isointense on T1WI, with variable signal intensity on T2WI. On contrast-enhanced MRI scans, they display mild hypointensity or isointensity in comparison with normal pituitary tissue^(6)^.

Pituitary macroadenomas exhibit a diverse enhancement pattern, indicating varying degrees of necrosis, cyst formation, and hemorrhage^(15)^. The enhancing portion of the tumors is considered to be the solid portion, and the cystic portions are defined as homogeneous, non-enhancing, sharply delineated areas on MRI^(12)^.

Adenomas with uncommon imaging characteristics account for approximately 5-15% of all PitNETs, with a significant proportion being pituitary macroadenomas^(7,16)^. In a study conducted by Zada et al.^(7)^, 18 atypical tumors were identified and 83% of those tumors were considered invasive on MRI, compared with 45% of those in the typical adenoma group. Such atypical adenomas are more common in female patients, among whom the tumors tend to be larger^(17)^, which may cause the lesion to appear heterogeneous, including calcifications (rare, found in 1-8% of cases), cyst formation/necrosis, and hemorrhage^(6)^, as well as lobulated configurations and marked invasion^(18,19)^.

The progression or recurrence of nonfunctioning pituitary macroadenomas can be predicted by DWI and apparent diffusion coefficient (ADC) values. Lower ADC values are associated with a higher risk of recurrence^(20)^. One application of ADC measurement in pituitary macroadenomas is to predict tumor consistency, a topic that remains a subject of debate. One recent study found that the effect of ADC on predictions is nonlinear, influenced by factors that can support or challenge the classification of tumor consistency as non-soft^(21)^. For example, larger tumors may exhibit more varied regions and levels of fibrosis, resulting in different behaviors in this characteristic. Therefore, the relationship may appear linear in smaller, more uniform specimens.

**Figure 1:**
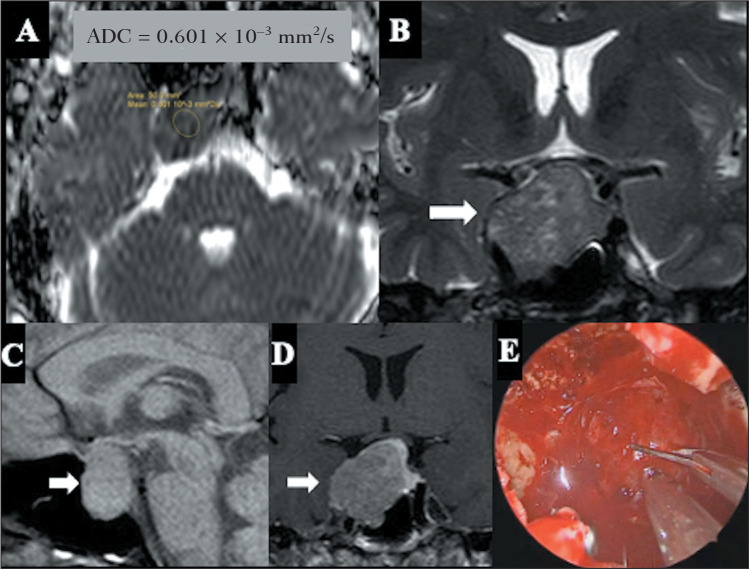
Surgically confirmed non-soft pituitary macroadenoma with a Ki-67 proliferation index of 1%. A: Axial ADC map showing a lesion coefficient of 0.60-10.3 mm^2^/s. Coronal T2WI (B), sagittal T1WI (C), and coronal T1WI after gadolinium (D), showing a solid, slightly heterogeneous, expansile sellar lesion with suprasellar extension and invasion of the right cavernous sinus, with hypoenhancement by contrast medium. E: Intraoperative picture of the endoscopic endonasal approach to pituitary macroadenoma-the fibroelastic consistency of the adenoma required sharp dissection with endonasal microscissors for the surgical resection.

On DWI, a less frequently observed pattern of pituitary macroadenomas is one of lesions showing ADC values of 0.5-1.0 × 10^-3^ mm^2^/s. That ADC range has been linked to a higher probability that a tumor will have a non-soft consistency, as illustrated in the probability contour maps generated by the machine learning model. This imaging phenotype may indicate increased tissue cellularity or reduced extracellular space, features commonly associated with fibrous adenomas. However, the relationship between the ADC and tumor consistency appears to be nonlinear and may be influenced by other factors, such as tumor diameter, patient age, and patient sex. The heterogeneous nature of pituitary macroadenomas, including the coexistence of fibrotic and nonfibrotic areas (Figure 1), may account for the variability in ADC behavior, supporting the idea that ADC alone is inadequate to predict consistency with high reliability^(21)^.

The consistency of a tumor, whether soft or non-soft, significantly influences surgical planning and requires skilled handling of various surgical tools. Soft tumors can often be removed easily using methods like aspiration or blunt curettage. However, approximately 10-15% of tumors are non-soft, posing significant technical challenges, with lower rates of complete resection and a higher risk of postoperative pituitary dysfunction^(22)^. In these situations, sharp instruments, curettage, ultrasonic aspirators, increased surgical access, or even craniotomy may be necessary^(23)^. Advanced MRI techniques, such as dynamic contrast-enhanced imaging with pharmacokinetic modeling, have shown potential for preoperative consistency assessment. These tools may assist neurosurgeons in anticipating surgical challenges, improving preoperative counseling, and guiding individualized strategies for tumor removal^(24)^.

The prognosis for pituitary macroadenomas with atypical features is variable, with some studies indicating a higher likelihood of recurrence and progression compared to typical adenomas^(16)^. However, the lack of standardized diagnostic criteria has historically made it challenging to predict outcomes accurately^(16)^. Nevertheless, it is essential to consider some radiological findings that favor the categorization of a pituitary macroadenoma as atypical.

### Less common imaging features of pituitary macroadenomas

#### Hemorrhages and areas of necrosis

**Figure 2:**
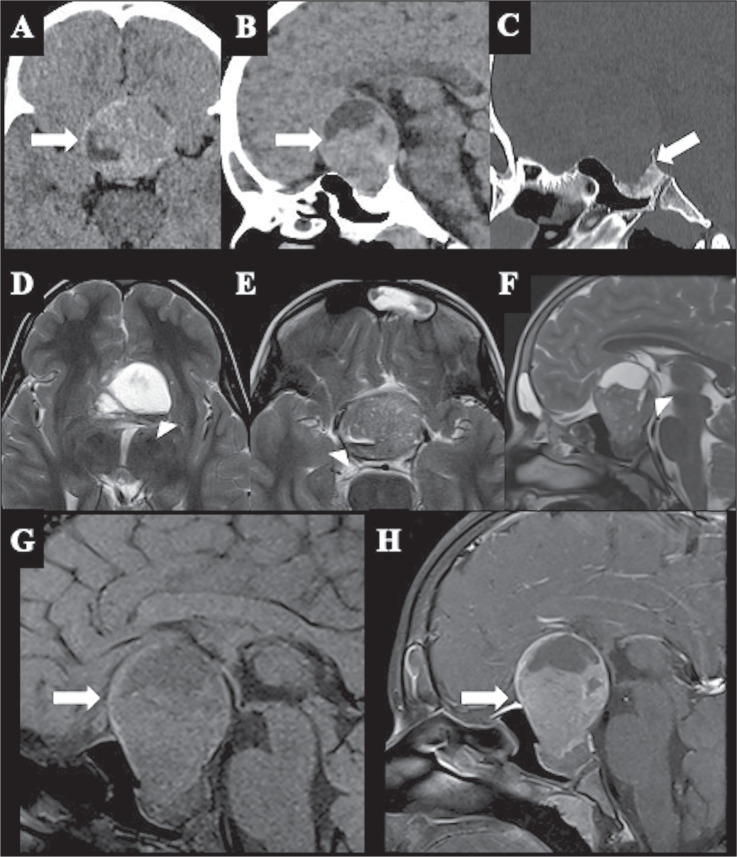
Pituitary macroadenoma with hematic component in a child. Axial and sagittal CT sequences (A and B, respectively) showing a heterogeneous, expansile lesion with solid and cystic components. Sagittal CT with bone window settings (C) showing sellar enlargement, without bone destruction. Note the presence of a fluid-fluid component, evidenced as hyperdensity on CT (A) and as hypointensity on MRI (T2WI, arrowheads in D, E, and F), which corresponds to the hematic component. Sagittal T1WI (G) and T1WI after gadolinium administration (H) showing enhancement in the solid components of the lesion.

Intratumoral hemorrhage and ischemic infarction are common in larger PitNETs, which may result in hemorrhagic changes, cystic changes, or both, leading to various signal intensities on MRI^(25)^. Pituitary macroadenomas often have a complex internal structure with varying degrees of necrosis and cystic changes, which can alter the typical progression of hemoglobin degradation^(26)^. The presence of necrotic tissue can affect the breakdown and absorption of blood products, leading to atypical imaging findings^(27)^. Advanced MRI techniques capable of detecting an adenoma and its hemorrhagic changes, such as T2*WI gradient-echo sequences and phase-sensitive imaging, reveal that hemorrhages in pituitary macroadenomas can present with diverse appearances, such as “rim”, “mass”, “spot”, and “diffuse” patterns (Figure 2), which do not necessarily correlate with the standard phases of hemoglobin degradation^(26,28)^.

Hemorrhage occurs in up to 25% of pituitary tumors. In cases of hemorrhage, the hematic components appear as hyperintense on T1WI, hypointense on T2WI, hypointense on T2*WI, and hypointense on SWI^(6)^. It is clinically asymptomatic in most patients and can be focal or diffuse throughout the adenoma. There is also restricted diffusion due to the accumulation of intracellular hemorrhagic products^(6)^.

A fluid-fluid level within the adenoma is highly suggestive of hemorrhage, which may occur at a later stage due to the sedimentation of blood products. The finding also aids in the differential diagnosis of craniopharyngioma, which may exhibit a hyperintense signal on T1WI because of the presence of many proteins. However, a craniopharyngioma less commonly presents with hemorrhage and therefore does not typically exhibit a fluid-fluid level. Differentiating necrosis from cystic changes due to previous hemorrhage in the adenoma is not possible by CT, but rather by MRI, which has high sensitivity when a spin-echo pulse sequence with a short repetition time/echo time (TR/TE) is used. In such sequences, the cystic areas will be predominantly hypointense, with the signal intensity increasing in sequences with longer TR/TE values. Areas with subacute and chronic hematic content show high signal intensity on spin-echo sequences with short TR/TE values^(29)^. Although CT has no role in evaluating previous and subacute hemorrhage, it can aid in assessing acute bleeding^(29)^, especially within the first 24-48 h, when it can reveal hyperdensity (60-90 HU).

An important differential diagnosis when identifying intratumoral hemorrhage is pituitary apoplexy, which corresponds to a rare yet potentially life-threatening clinical syndrome characterized by the sudden onset of symptoms such as severe headache, vomiting, visual disturbances, ophthalmoplegia, altered mental status, and possible panhypopituitarism. This condition predominantly occurs in individuals with hemorrhagic infarction of the pituitary gland, often associated with an existing pituitary macroadenoma. While certain pathological and physiological conditions may present with similar imaging features, a combination of clinical presentation and imaging characteristics can help radiologists arrive at an accurate diagnosis, particularly through the use of MRI^(14)^.

**Figure 3:**
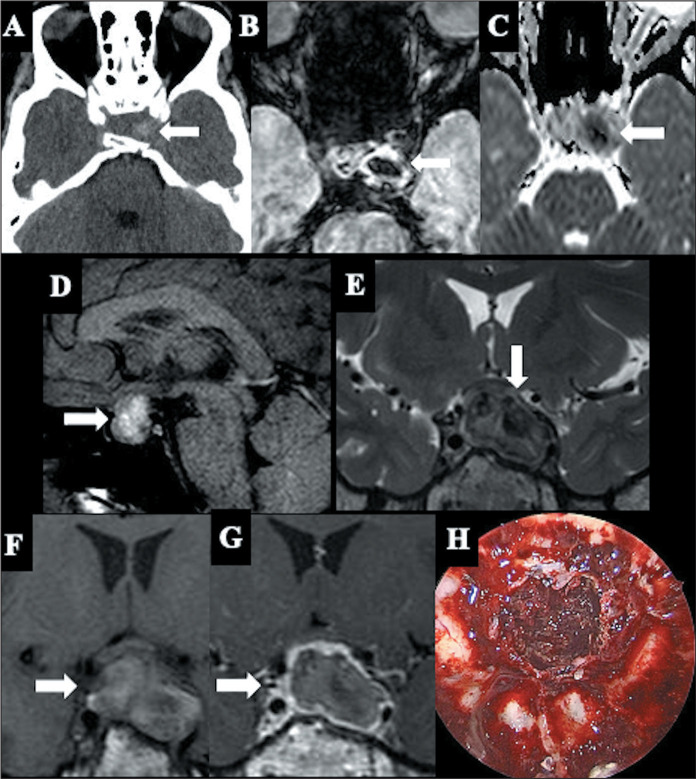
Pituitary apoplexy. A: Axial CT scan showing a hyperdense area (arrow), corresponding to hypointensity on SWI (B), restricted diffusion on the ADC map (C), and hyperintensity on a sagittal T1WI sequence (D). Coronal T2WI (E) showing areas of hypointensity. T1WI (F) and T1WI after gadolinium administration (G) showing hypoenhancement of the lesion. H: Intraoperative photograph of the endoscopic endonasal approach to a pituitary macroadenoma in an apoplexy scenario, demonstrating the hemorrhagic appearance of the adenoma.

Pituitary apoplexy can present with hemorrhagic features that are sometimes detectable on CT scans as hyperdense areas within the lesion, although similar findings can occur in other conditions. The accuracy of CT is variable, depending on factors such as timing after symptom onset and image quality, particularly as blood products evolve over time. Following the administration of intravenous contrast, the presence of an enhancement rim may indicate pituitary apoplexy. However, MRI is the primary diagnostic modality for pituitary apoplexy, capable of identifying hemorrhagic changes in adenomas. On T1WI, such changes appear as hyperintense areas, and gadolinium-enhanced images reveal subtle, uneven enhancement. On T2WI, mixed signal intensities and a peripheral hypointense rim due to hemosiderin deposition are often seen, which helps assess compression of adjacent structures, such as the optic chiasm and hypothalamus (Figure 3). A highly specific MRI sign of acute pituitary apoplexy is mucosal thickening in the sphenoid sinus, likely related to venous congestion, which may resolve over time^(14)^.

In most cases of prolactinomas, the use of dopaminergic agonists induces significant tumor regression and may therefore lead to pituitary apoplexy. Although most of the cases described occurred with the use of bromocriptine, cases associated with the use of cabergoline have also been reported^(30,31)^. It is worth noting that even in cases of apoplexy, therapy to control prolactin levels and tumor size should be continued. Pituitary apoplexy has also been described in patients with a prolactinoma during pregnancy, regardless of tumor size^(32-34)^.

The first case of pituitary apoplexy described in the literature was in a patient with a growth hormone (GH)-producing pituitary tumor. Apoplexy can occur in these tumors with variable outcomes, ranging from fulminant complications to complete hormonal remission, and may induce partial or complete hypopituitarism, including vasopressin deficiency^(35,36)^. In these cases, apoplexy is more common if the tumor is invasive, can occur at any age, and has no predilection for either sex.

Among patients with nonfunctioning PitNETs, the estimated event rate for stroke is 0.2-0.6 per 100 person-years^(37,38)^. It is known that 45% of all PitNETs that develop pituitary apoplexy are nonfunctioning^(39)^. Although apoplexy occurs most commonly in pituitary macroadenomas, it has also been reported in nonfunctioning pituitary microadenomas^(40,41)^.

The main cause of spontaneous remission of Cushing’s disease is pituitary tumor apoplexy^(42)^, however tumor recurrence after pituitary apoplexy has also been described, justifying the need for prolonged monitoring in these patients^(43)^.

#### Cystic components

Pituitary macroadenomas frequently appear as cystic masses. When a cystic mass in the sellar region shows a fluid-fluid level, it is usually indicative of a cystic PitNET^(5,29)^.

Cystic lesions of the sellar region are considered to be any predominantly fluid-containing lesion. The type of fluid can vary, including cerebrospinal fluid, bleeding, necrotic fluid, fluid with a high protein content, or oily contents. On MRI, such fluid contents typically present as nonenhancing components on contrast-enhanced T1WI sequences. There can be enhancement around such fluid content, either in the cyst/tumor walls or in the remnant of the pituitary gland^(44)^. Cystic areas in pituitary macroadenomas may be sequelae of previous focal infarction or intratumoral hemorrhage^(29)^.

When the entire lesion presents with either hemorrhage or cystic/necrotic degeneration, pituitary macroadenomas appear as cystic sellar or suprasellar lesions. Thick wall enhancement and internal septation represent nondegenerated tumor fragments or walls^(25)^. The preserved pituitary parenchyma can sometimes be identified as a solid enhancing area in the periphery of the tumor, an off-midline location. It has also been suggested that lateral bulging of the pituitary gland and displacement of the pituitary stalk favor a diagnosis of cystic adenoma^(12,44)^.

A recent review and meta-analysis showed that 60% of cystic adenomas are prolactin-producing, whereas 26% are nonfunctioning and 9% are GH-producing. When compared with solid adenomas, cystic tumors have the same postoperative rates of recurrence and hormonal remission^(45)^.

**Figure 4:**
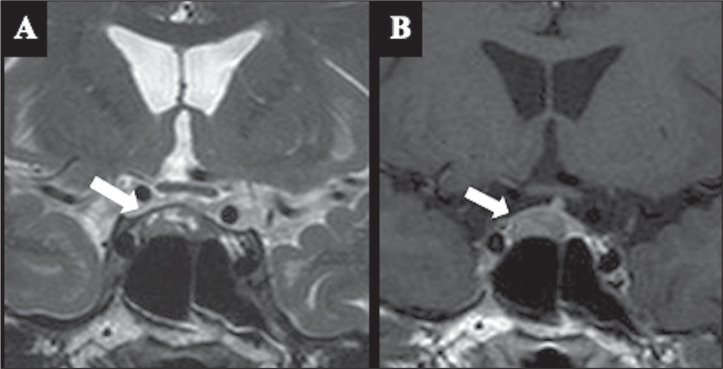
Confirmed corticotroph PitNET. T2WI (A) showing small intralesional cysts, and T1WI after gadolinium administration (B) showing hypoenhancement.

Silent corticotroph PitNETs typically present as macroadenomas and are more aggressive than other nonfunctioning types. A finding of multiple cysts on T2WI strongly suggests this subtype, with a sensitivity of 58% and specificity of 93% (Figure 4). These tumors show higher rates of preoperative hypopituitarism, cavernous sinus invasion, and early recurrence^(46)^.

Rathke’s cleft cysts (RCCs) are benign midline epithelial cysts that may closely mimic cystic PitNETs due to variable signal intensities on MRI and typically present as midline, nonenhancing, noncalcified intrasellar or suprasellar cysts, often containing an intracystic nodule, which is considered the most specific imaging finding. However, because of their variable signal intensities on T1WI and T2WI, particularly when protein-rich content leads to hyperintensity and hypointensity, respectively, RCCs can closely mimic cystic PitNETs, especially those with hemorrhage^(25)^. Certain imaging features, such as fluid-fluid levels, septations, and lateral or off-midline location, favor a diagnosis of cystic PitNET. In contrast, a midline cyst with an intracystic hypointense nodule strongly suggests an RCC. In addition, adenomas generally arise from the adenohypophysis and may demonstrate lateral bulging of the gland with infundibular displacement, features not typical of an RCC^(47)^.

#### Calcifications

Intracranial calcifications seen on CT may be physiological, age-related, or pathological and are particularly important in narrowing the differential diagnosis of suprasellar lesions. They are easily recognizable on CT because of their high attenuation values, exceeding 100 HU. On MRI, signal intensity on T1WI or T2WI spin-echo sequences is variable and nonspecific for identifying calcifications. On gradient-echo sequences and SWI, intracranial calcifications and hemorrhages both appear hypointense. The SWI utilizes a phase map, which is crucial in differentiating between the two etiologies, because calcifications are diamagnetic and iron is paramagnetic, resulting in opposite signal intensities^(48)^. However, it should be borne in mind that the use of SWI in the sellar region is often limited due to susceptibility artifacts at the skull base, particularly in lesions with minimal suprasellar extension.

Although PitNETs rarely exhibit calcification radiologically (in only 0.2-8.0% of cases), histology reveals psammoma bodies in 15-25% of cases. Calcifications in adenomas typically appear as a thin peripheral “eggshell” layer or scattered internal nodules, with the term “pituitary stone” describing a large, dense calcified deposit^(49)^. Calcification in the sellar region typically favors alternative diagnoses, such as craniopharyngioma, meningioma, or aneurysm, over that of PitNET^(50)^.

Although most cases of calcifications in PitNETs have been described in functioning tumors, especially prolactinomas, nonfunctioning adenomas may also present calcifications. In those cases, the differential diagnosis with other lesions of the sellar region, such as craniopharyngiomas, is more difficult because of the absence of symptoms of hormonal excess^(51)^.

#### Extrasellar location

Ectopic PitNETs are rare tumors located outside the sella turcica, without any connections to the intrasellar components^(52)^. The most common sites are the sphenoid sinus (in 34.4%) and the suprasellar region (in 25.6%), with less common locations including the clivus, cavernous sinus, and nasopharynx. Rarer sites include the nasal cavity, orbital structures, and paranasal sinuses. Ectopic PitNETs can invade adjacent structures, particularly those in the clivus and sphenoid sinus, where bone involvement is common. Some large sphenoid sinus tumors may extend to the sphenoid wing, petrosal bone tip, and even the inner temporal bone. In contrast, large clival lesions may reach the superior orbital fissure, internal auditory canals, and nasopharynx. In comparison, nasopharyngeal PitNETs typically show minimal invasion^(53)^.

In a review of 180 patients with ectopic PitNETs, the mean age at diagnosis was 45.4 years. They were located mainly in the sphenoid sinus (in 34.4%) and suprasellar region (in 25.6%), followed by the clivus (in 15.6%), cavernous sinus (in 13.3%), and nasopharynx (in 5.6%). Although most of the adenomas were adrenocorticotropic hormone-secreting and nonfunctioning (38.9% and 27.2%, respectively), prolactinomas have also been described, as have GH- and thyroid-stimulating factor-producing adenomas, the latter being more common in tumors located in the nasopharynx. In patients with suprasellar tumors, the most common complaints were menstrual disturbances and visual alterations, whereas headache was the main complaint in those with clival tumors. It is worth noting that most adenomas located in the suprasellar space and cavernous sinus were diagnosed by imaging examinations and can be confused with sellar tumors given the anatomical proximity^(54)^.

Clinical characteristics vary between tumors at different locations. Patients with ectopic PitNETs require hormonal investigation by a specialist and careful evaluation of imaging examinations, including, if necessary, nuclear medicine imaging examinations. When the diagnosis is not well established, biopsy of the lesion may be considered for differential diagnosis, provided that it will not interfere with the treatment of the patient, and hypophysectomy should not be performed when the tumor is not well located. The diagnosis and treatment of these patients should be personalized according to the location of and hormones secreted by the tumor.

#### Bone invasion

The sella is a concavity in the midline of the base of the sphenoid where the pituitary gland is located, limited anteriorly by the anterior clinoid processes of the lesser wing of the sphenoid, posteriorly by the dorsum of the sella, and inferiorly by the roof of the sphenoid sinus^(55)^. Typically, pituitary macroadenomas lead to enlargement and remodeling of the sella. If invasive or large, they cause erosion and bone destruction, invading the base of the skull in some cases, thus simulating infectious processes or metastases^(11)^.

Bone-invasive PitNETs are a subtype of pituitary macroadenoma, with aggressive behavior, posing significant surgical challenges due to invasion of critical bone structures. These tumors fragment normal bone, increasing the risk of damaging vital vessels, cranial nerves, or brain tissue during surgery. Extensive bone invasion complicates complete resection and raises the likelihood of recurrence. Comprehensive preoperative and postoperative imaging, including CT-sellar, nasal, and three-dimensional (3D) reconstruction-and MRI-T1WI, T2WI, and contrast-enhanced sequences-is essential for assessing tumor extent and bone involvement. Specialized MRI sequences, like 3D fast imaging employing steady-state acquisition (3D-FIESTA), 3D constructive interference in steady state (3D-CISS), and 3D sampling perfection with application-optimized contrasts using different flip angle evolution (3D-SPACE), may help detect oculomotor nerve compression^(53)^.

A study involving 107 patients with bone-invasive adenomas demonstrated, through hormonal evaluation, that the majority were nonfunctioning tumors, followed by tumors producing GH and prolactin^(53)^. It is known that silent corticotroph adenomas, in comparison with nonfunctioning adenomas, are more invasive in bone structures, more commonly grow through the sellar floor, and are more likely to invade the sphenoid sinus. In addition, they are more likely to invade the clivus posteroinferiorly and the cavernous sinuses laterally than are nonfunctioning adenomas, which generally grow toward the region with least resistance (i.e., the suprasellar space). Bone invasion makes the surgical treatment of these adenomas more challenging (Table 1), which explains the higher recurrence rates among silent corticotroph adenomas^(56)^.

**Table 1:** Typical versus atypical pituitary macroadenomas.

Pattern of pituitary macroadenomas	Imaging findings	Differential diagnoses	Clinical implications
Typical	Snowman-like or ovoid shape; isointense/ slightly hypointense on T1WI; variable intensity on T2WI; homogeneous or heterogeneous enhancement; sellar enlargement without marked invasion	Craniopharyngioma and meningioma (rare overlap with typical appearance)	Usually predictable surgical resection; low recurrence rates; minimally aggressive clinical course
Atypical Cystic variants and hemorrhages	Predominantly cystic content; fluid-fluid levels, septations; wall or rim enhancement; possible hemorrhagic or necrotic components	RCC (intracystic midline nodule), craniopharyngioma (protein-rich cysts), and arachnoid cyst	Diagnosis impacts surgical planning; differentiation avoids unnecessary surgery in RCC; prognosis similar to that of solid adenomas when correctly managed
Calcified variants	Thin peripheral or nodular calcifications (rare, 0.2-8.0% of cases); better visualized on CT; hypointense on GRE/SWI	Craniopharyngioma, meningioma, and aneurysm	Calcification suggests alternative diagnoses; most often associated with functioning adenomas
Bone-invasive adenomas	Sellar erosion, with clival or sphenoid sinus invasion; fragmentation of normal bone on CT and MRI	Infectious processes, metastases, meningioma, and chordoma	Surgical challenge due to bone involvement; higher recurrence rates; increased risk of vascular/cranial nerve injury

### Applicability of additional MRI sequences for better evaluation of pituitary macroadenomas

#### 3D-CISS

An additional gradient-echo MRI sequence used in order to enhance the evaluation of certain anatomical information is 3D-CISS, which provides greater sensitivity because the accentuation of T2WI values between the cerebrospinal fluid and pathological structures. In the case of sellar lesions, such as pituitary macroadenomas, 3D-CISS can be used to optimize the assessment of the extent of the tumor and its relationship with the cavernous sinuses, being useful for detecting subtle lesions that may not be visible in routine spin-echo sequences^(57)^.

#### Contrast-enhanced volumetric sequences

Contrast-enhanced T1WI volumetric 3D sequences, such as fast spoiled gradient-recalled (SPGR) and fast spin-echo techniques-SPACE, volume isotropic turbo spin-echo acquisition (VISTA), and the VISTA analogue CUBE-play fundamental roles in the MRI protocol for pituitary macroadenomas. These sequences enable high-resolution isotropic imaging with multiplanar reconstruction, which is particularly important for evaluating the relationship of the lesion with critical adjacent structures, including the optic chiasm, optic nerves, and cavernous sinus. Compared with traditional 2D T1-weighted spin-echo imaging, volumetric acquisitions provide superior spatial resolution and contrast-to-noise ratio, improving the accuracy of lesion characterization^(58)^.

Another advantage of 3D T1-weighted volumetric imaging is that it can reduce partial volume effects and improve the delineation of tumor margins, especially for tumors with atypical growth patterns. In addition, the ability to reconstruct images in multiple planes from a single acquisition facilitates preoperative assessment and surgical planning, because it allows precise evaluation of the extent of the tumor and its connection to neurovascular structures^(55)^. These characteristics make contrast-enhanced volumetric sequences an essential component of modern MRI protocols for pituitary macroadenomas, increasing diagnostic confidence and informing clinical decision-making.

#### Spectroscopy

Proton magnetic resonance spectroscopy (^1^H-MRS) is a valuable tool for studying pituitary macroadenomas, providing insights into their metabolic profiles and potential alterations. It is particularly useful for assessing proliferative potential and identifying specific metabolic markers. It also provides a noninvasive method for monitoring structural and metabolic changes in pituitary macroadenomas, aiding in the assessment of tumor proliferation and hemorrhagic status^(59)^.

Elevated levels of choline-containing compounds are often observed in pituitary macroadenomas, correlating with increased cell proliferation. This is evidenced by a strong positive correlation between choline levels and the MIB-1 proliferative cell index in nonhemorrhagic pituitary macroadenomas^(59,60)^. High choline peaks can indicate active cell proliferation and hormonal activity in functional adenomas^(60)^.

In cases of hemorrhagic adenomas, there is typically no assignable concentration of choline, and the full width at half maximum of the water peak is increased, likely due to the fact that the presence of iron ions from hemosiderin decreases magnetic field homogeneity^(59)^.

In patients with succinate dehydrogenase gene mutations, ^1^H-MRS can detect succinate at 2.4 ppm as a biomarker, indicating succinate dehydrogenase deficiency. This is particularly relevant in macroprolactinomas and pheochromocytomas/paragangliomas, for which succinate peaks are observed, linking succinate dehydrogenase mutations to tumor development^(61)^. In addition, ^1^H-MRS can assist in differentiating between various types of brain tumors by identifying unique metabolic profiles, such as elevated choline in PitNETs and other specific metabolites in different tumor types^(59)^.

#### Perfusion

Perfusion MRI, particularly that involving techniques such as arterial spin labeling (ASL) and perfusion-weighted imaging, is valuable for studying pituitary macroadenomas. These techniques help assess vascular characteristics and differentiate between different types of tumors.

The ASL technique is effective in reflecting the vascular density of nonfunctioning pituitary macroadenomas. Unlike routine contrast enhancement, ASL correlates with microvascular density. This correlation can help predict the risks of intraoperative and postoperative hemorrhage^(62)^. Sakai et al.^(62)^ found that ASL perfusion imaging results correlated with the histologic total microvascular density of pituitary macroadenomas. This suggests that ASL can effectively reflect the angiogenic activity within these tumors, a critical factor in determining their grade and potential behavior. The authors detected no correlation between routine contrast enhancement and microvascular density. However, they found that cerebral blood flow measured by ASL did correlate with microvascular density, indicating that ASL reflects the vascular characteristics of a tumor more accurately than does traditional contrast-enhanced MRI^(62)^.

Perfusion-weighted imaging, including signal-intensity curve analysis, aids in the differential diagnosis of sellar and parasellar tumors. It distinguishes between high-perfusion tumors like pituitary macroadenomas and meningiomas and low-perfusion neoplasms such as adamantinomatous craniopharyngiomas. Significant differences in relative cerebral blood volume values help distinguish adenomas from other tumors^(63)^. Perfusion-weighted imaging provides additional diagnostic information, making it a useful tool in differentiating between various types of sellar and parasellar tumors, which often appear similar on plain MRI^(63)^.

Despite their potential to provide additional metabolic and hemodynamic information, ^1^H-MRS and perfusion-weighted imaging are of limited practical utility in pituitary macroadenomas. These techniques are not routinely incorporated into standard MRI protocols and are generally reserved for selected cases, such as tumors associated with genetic syndromes or when differential diagnosis with other sellar or parasellar lesions is required.

### Pitfall and differential diagnosis

#### Pituitary carcinoma

Metastatic PitNETs, as pituitary carcinomas are currently referred to in the 5th edition of the WHO classification^(5)^, are rare pituitary neuroendocrine tumors that spread to lymph nodes, distant organs, or via discontinuous central nervous system dissemination, accounting for only 0.1% of all pituitary tumors^(64)^. Previously classified as “pituitary carcinomas” in earlier WHO editions, the 5th edition of the WHO classification now designates all PitNETs as malignant, using the term metastatic PitNETs for those with confirmed metastases^(5)^. These lesions are histologically indistinguishable from typical PitNETs. Most originate as invasive lactotroph or corticotroph tumors and subsequently metastasize, in which cases the average survival is less than four years^(65)^.

**Figure 5:**
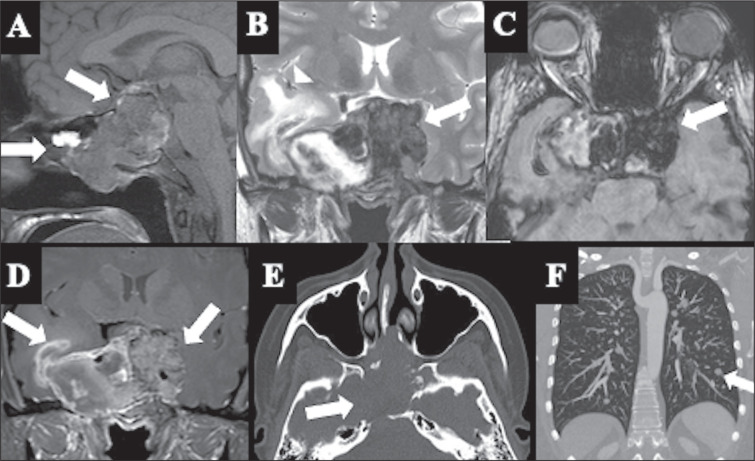
Metastatic PitNET. Solid, heterogeneous, lobulated expansile lesion, extending into the suprasellar region and the sphenoid sinus, with hematic components, characterized by hyperintensity on sagittal T1WI (A), hypointensity on coronal T2WI (B) and hypointensity on axial SWI (C). Note the lesion invading the bilateral cavernous sinuses and the right temporal lobe, with associated vasogenic edema (arrowhead in B). D: T1WI after gadolinium administration showing intense heterogeneous contrast enhancement. E: CT with bone window settings showing bone destruction of the skull base. F: Lung CT, with maximum intensity projection reconstruction, showing multiple bilateral pulmonary nodules, consistent with distant metastases.

Some pituitary tumors destined to become carcinomas show early aggressive behavior (Figure 5), with rapid recurrence and progression after initial surgery, while others evolve into carcinomas only after many years. Notably, pituitary carcinoma can remain undetected for a long time despite persistently elevated biochemical markers and no visible sellar tumor, as illustrated by cases in which distant metastases, such as GH-immunopositive cervical adenopathy, reveal the diagnosis^(64)^.

## CONCLUSION

Although the atypical aspects of pituitary macroadenomas on various imaging methods, such as hemorrhages, necrotic/cystic areas, extrasellar location, and bone invasion, are less common, considering such aspects is crucial for raising diagnostic hypotheses and thereby facilitating a correct diagnosis. However, we must keep in mind that no imaging aspect is pathognomonic; therefore, it is essential also to understand the main diagnostic differences and their characteristics. The correlations between histological and imaging data underscore the importance of integrating all available data for the accurate diagnosis and appropriate management of these tumors.

## Data Availability

Not applicable.

## References

[1] Gupta K, Sahni S, Saggar K (2018). Evaluation of clinical and magnetic resonance imaging profile of
pituitary macroadenoma: a prospective study. J Nat Sci Biol Med.

[2] Osborn AG, Louis DN, Poussaint TY (2022). The 2021 World Health Organization classification of tumors of
the central nervous system: what neuroradiologists need to
know. AJNR Am J Neuroradiol.

[3] Tahara S, Hattori Y, Suzuki K (2022). An overview of pituitary incidentalomas: diagnosis, clinical
features, and management. Cancers (Basel).

[4] Pisaneschi M, Kapoor G. (2005). Imaging the sella and parasellar region. Neuroimaging Clin N Am.

[5] Tsukamoto T, Miki Y. (2023). Imaging of pituitary tumors: an update with the 5th WHO
Classifications - part 1. Pituitary neuroendocrine tumor (PitNET)/pituitary
adenoma. Jpn J Radiol.

[6] Ouyang T, Rothfus WE, Ng JM (2011). Imaging of the pituitary. Radiol Clin North Am.

[7] Zada G, Woodmansee WW, Ramkissoon S (2011). Atypical pituitary adenomas: incidence, clinical characteristics,
and implications. J Neurosurg.

[8] Zada G, Lin N, Laws ER (2010). Patterns of extrasellar extension in growth hormone-secreting and
nonfunctional pituitary macroadenomas. Neurosurg Focus.

[9] Hladik M, Nasi-Kordhishti I, Dörner L (2024). Comparative analysis of intraoperative and imaging features of
invasive growth in pituitary adenomas. Eur J Endocrinol.

[10] Silva NA, Reis F, Miura LK (2014). Pituitary macroadenoma presenting as a nasal tumor: case
report. Sao Paulo Med J.

[11] Shih RY, Schroeder JW, Koeller KK. (2021). Primary tumors of the pituitary gland: radiologic-pathologic
correlation. Radiographics.

[12] Choi SH, Kwon BJ, Na DG (2007). Pituitary adenoma, craniopharyngioma, and Rathke cleft cyst
involving both intrasellar and suprasellar regions: differentiation using
MRI. Clin Radiol.

[13] Colégio Brasileiro de Radiologia e Diagnóstico por
Imagem CBR Clínicas. Protocolos iniciais de ressonância magnética.

[14] Boellis A, di Napoli A, Romano A (2014). Pituitary apoplexy: an update on clinical and imaging
features. Insights Imaging.

[15] Pantalone KM, Jones SE, Weil RJ, Pantalone KM, Jones SE, Weil RJ (2015). MRI atlas of pituitary pathology.

[16] Chesney K, Memel Z, Pangal DJ (2018). Variability and lack of prognostic value associated with atypical
pituitary adenoma diagnosis: a systematic review and critical assessment of
the diagnostic criteria. Neurosurgery.

[17] Rutkowski MJ, Alward RM, Chen B (2018). Atypical pituitary adenoma: a clinicopathologic case
series. J Neurosurg.

[18] Nishioka H, Inoshita N, Sano T (2012). Correlation between histological subtypes and MRI findings in
clinically nonfunctioning pituitary adenomas. Endocr Pathol.

[19] Bette S, Butenschön VM, Wiestler B (2020). MRI criteria of subtypes of adenomas and epithelial cysts of the
pituitary gland. Neurosurg Rev.

[20] Ko CC, Chen TY, Lim SW (2020). Prediction of recurrence in solid nonfunctioning pituitary
macroadenomas: additional benefits of diffusion-weighted MR
imaging. J Neurosurg.

[21] Pereira FV, Ferreira D, Garmes R (2025). Machine learning prediction of pituitary macroadenoma
consistency: utilizing demographic data and brain MRI
parameters. J Imaging Inform Med.

[22] Kamimura K, Nakajo M, Bohara M (2021). Consistency of pituitary adenoma: prediction by pharmacokinetic
dynamic contrast-enhanced MRI and comparison with histologic collagen
content. Cancers (Basel).

[23] Acitores Cancela A, Rodríguez Berrocal V, Pian Arias H (2022). Effect of pituitary adenoma consistency on surgical outcomes in
patients undergoing endonasal endoscopic transsphenoidal
surgery. Endocrine.

[24] De Alcubierre D, Puliani G, Cozzolino A (2023). Pituitary adenoma consistency affects postoperative hormone
function: a retrospective study. BMC Endocr Disord.

[25] Park M, Lee SK, Choi J (2015). Differentiation between cystic pituitary adenomas and Rathke
cleft cysts: a diagnostic model using MRI. AJNR Am J Neuroradiol.

[26] Tosaka M, Sato N, Hirato J (2007). Assessment of hemorrhage in pituitary macroadenoma by
T2*-weighted gradient-echo MR imaging. AJNR Am J Neuroradiol.

[27] Lazaro CM, Guo WY, Sami M (1994). Haemorrhagic pituitary tumours. Neuroradiology.

[28] Kurosaki M, Tabuchi S, Akatsuka K (2010). Application of phase sensitive imaging (PSI) for hemorrhage
diagnosis in pituitary adenomas. Neurol Res.

[29] Ostrov SG, Quencer RM, Hoffman JC (1989). Hemorrhage within pituitary adenomas: how often associated with
pituitary apoplexy syndrome?. AJR Am J Roentgenol.

[30] Chng E, Dalan R. (2013). Pituitary apoplexy associated with cabergoline
therapy. J Clin Neurosci.

[31] Lima GAB, Machado EO, Silva CMS (2008). Pituitary apoplexy during treatment of cystic macroprolactinomas
with cabergoline. Pituitary.

[32] Barraud S, Guédra L, Delemer B (2020). Evolution of macroprolactinomas during pregnancy: a cohort study
of 85 pregnancies. Clin Endocrinol.

[33] Kuhn E, Weinreich AA, Biermasz NR (2021). Apoplexy of microprolactinomas during pregnancy: report of five
cases and review of the literature. Eur J Endocrinol.

[34] Khaldi S, Saad G, Elfekih H (2021). Pituitary apoplexy of a giant prolactinoma during
pregnancy. Gynecol Endocrinol.

[35] Zheng XQ, Zhou X, Yao Y (2023). Acromegaly complicated with fulminant pituitary apoplexy:
clinical characteristic analysis and review of literature. Endocrine.

[36] Fraser LA, Lee D, Cooper P (2009). Remission of acromegaly after pituitary apoplexy: case report and
review of literature. Endocr Pract.

[37] Fernández-Balsells MM, Murad MH, Barwise A (2011). Natural history of nonfunctioning pituitary adenomas and
incidentalomas: a systematic review and metaanalysis. J Clin Endocrinol Metab.

[38] Sivakumar W, Chamoun R, Nguyen V (2011). Incidental pituitary adenomas. Neurosurg Focus.

[39] Nielsen EH, Lindholm J, Bjerre P (2006). Frequent occurrence of pituitary apoplexy in patients with
non-functioning pituitary adenoma. Clin Endocrinol.

[40] Hamblin R, Fountas A, Lithgow K (2023). Natural history of non-functioning pituitary microadenomas:
results from the UK non-functioning pituitary adenoma
consortium. Eur J Endocrinol.

[41] Pernik MN, Montgomery EY, Isa S (2022). The natural history of non-functioning pituitary adenomas: a
meta-analysis of conservatively managed tumors. J Clin Neurosci.

[42] Ilie IRP, Herdean AM, Herdean AI (2021). Spontaneous remission of Cushing’s disease: a systematic
review. Ann Endocrinol (Paris).

[43] Machado MC, Gadelha PS, Bronstein MD (2013). Spontaneous remission of hypercortisolism presumed due to
asymptomatic tumor apoplexy in ACTH-producing pituitary
macroadenoma. Arq Bras Endocrinol Metabol.

[44] Gadelha MR, Wildemberg LE, Lamback EB (2022). Approach to the patient: differential diagnosis of cystic sellar
lesions. J Clin Endocrinol Metabol.

[45] Webb KL, Hinkle ML, Walsh MT (2024). Surgical treatment of cystic pituitary adenomas: literature-based
definitions and postoperative outcomes. Pituitary.

[46] Kasuki L, Antunes X, Coelho MCA (2020). Accuracy of microcystic aspect on T2-weighted MRI for the
diagnosis of silent corticotroph adenomas. Clin Endocrinol.

[47] Wang SS, Xiao DY, Yu YH (2012). Diagnostic significance of intracystic nodules on MRI in Rathke’s
cleft cyst. Int J Endocrinol.

[48] Wu Z, Mittal S, Kish K (2009). Identification of calcification with MRI using
susceptibility-weighted imaging: a case study. J Magn Reson Imaging.

[49] Albadr FB, Alhatlani AH, Alhelal NS (2024). Calcified pituitary adenoma mimicking craniopharyngioma: a case
report. Cureus.

[50] Jipa A, Jain V. (2021). Imaging of the sellar and parasellar regions. Clin Imaging.

[51] Gokbel A, Uzuner A, Emengen A (2024). Endoscopic endonasal approach for calcified sellar/parasellar
region pathologies: report of 11 pituitary adenoma cases. World Neurosurg.

[52] Demir MK, Ertem Ö, Kılıç D (2024). Ectopic pituitary neuroendocrine tumors/adenomas around the sella
turcica. Balkan Med J.

[53] Zhu H, Li B, Li C (2021). The clinical features, recurrence risks and surgical strategies
of bone invasive pituitary adenomas. Clin Neurol Neurosurg.

[54] Zhu J, Wang Z, Zhang Y (2020). Ectopic pituitary adenomas: clinical features, diagnostic
challenges and management. Pituitary.

[55] Osborn AG. (2014). Porto Alegre.

[56] Himstead AS, Wells AC, Kurtz JS (2024). Silent corticotroph adenomas demonstrate predilection for
sphenoid sinus, cavernous sinus, and clival invasion compared with other
subtypes. World Neurosurg.

[57] Hingwala D, Chatterjee S, Kesavadas C (2011). Applications of 3D CISS sequence for problem solving in
neuroimaging. Indian J Radiol Imaging.

[58] Hasegawa H, Ashikaga R, Okajima K (2017). Comparison of lesion enhancement between BB Cube and 3D-SPGR
images for brain tumors with 1.5-T magnetic resonance
imaging. Jpn J Radiol.

[59] Stadlbauer A, Buchfelder M, Nimsky C (2008). Proton magnetic resonance spectroscopy in pituitary
macroadenomas: preliminary results. J Neurosurg.

[60] Kozić D, Medić-Stojanoska M, Ostojić J (2007). Application of MR spectroscopy and treatment approaches in a
patient with extrapituitary growth hormone secreting
macroadenoma. Neuro Endocrinol Lett.

[61] Branzoli F, Salgues B, Marjańska M (2023). SDHx mutation and pituitary adenoma: can in vivo 1H-MR
spectroscopy unravel the link?. Endocr Relat Cancer.

[62] Sakai N, Koizumi S, Yamashita S (2013). Arterial spin-labeled perfusion imaging reflects vascular density
in nonfunctioning pituitary macroadenomas. AJNR Am J Neuroradiol.

[63] Bladowska J, Zimny A, Guziński M (2013). Usefulness of perfusion weighted magnetic resonance imaging with
signal-intensity curves analysis in the differential diagnosis of sellar and
parasellar tumors: preliminary report. Eur J Radiol.

[64] Garmes HM, Carvalheira JBC, Reis F (2017). Pituitary carcinoma: a case report and discussion of potential
value of combined use of Ga-68 DOTATATE and F-18 FDG PET/CT scan to better
choose therapy. Surg Neurol Int.

[65] Majd N, Waguespack SG, Janku F (2020). Efficacy of pembrolizumab in patients with pituitary carcinoma:
report of four cases from a phase II study. J Immunother Cancer.

